# Evaluating Clinical Efficacy and Survival Outcomes of Crizotinib in Anaplastic Lymphoma Kinase (ALK)-Positive Non-small Cell Lung Cancer (NSCLC): A Systematic Review and Meta-Analysis

**DOI:** 10.7759/cureus.102697

**Published:** 2026-01-31

**Authors:** Hunter W Brady, Jasneet Gill, Jun Wang

**Affiliations:** 1 Medicine, Lincoln Memorial University-DeBusk College of Osteopathic Medicine, Knoxville, USA; 2 Internal Medicine, Tennova North Knoxville Medical Center, Knoxville, USA; 3 Pathology, Lincoln Memorial University-DeBusk College of Osteopathic Medicine, Knoxville, USA

**Keywords:** alk positive nsclc, alk rearrangement, crizotinib, lung cancer, meta analysis, objective response rate, overall survival, progression free survival, targeted therapy, tyrosine kinase inhibitor

## Abstract

Non-small cell lung cancer (NSCLC) remains a major contributor to cancer-related deaths globally. Advances in tumor molecular profiling have led to the identification of actionable oncogenic alterations, including rearrangements involving the anaplastic lymphoma kinase (ALK) gene, which occur in a distinct subset of NSCLC patients. Crizotinib, an early-generation ALK-targeted tyrosine kinase inhibitor, has been widely used in this population; however, its overall clinical benefit relative to conventional chemotherapy continues to warrant systematic evaluation. This study synthesized published clinical evidence to assess objective response rate (ORR), progression-free survival (PFS), and overall survival (OS) among patients with ALK-positive NSCLC treated with crizotinib. A meta-analysis incorporating 38 eligible studies retrieved from PubMed was conducted, with inclusion criteria based on comparable study design and outcome reporting. Statistical analyses were performed using Review Manager 5 (The Cochrane Collaboration, London, England, UK), and sensitivity analyses were undertaken following exclusion of studies deemed to have a high risk of bias. Survival outcomes were summarized using reported means and ranges across multiple follow-up intervals, and treatment effects were quantified using odds ratios (ORs) with corresponding p-values. Compared with chemotherapy, crizotinib treatment resulted in a markedly higher ORR (OR = 6.86; 95% CI, 4.39-10.73; p < 0.00001). Reported median PFS ranged from 6.8 to 19 months in the crizotinib cohorts, versus 2.4 to seven months among chemotherapy-treated patients. At six and 12 months, PFS rates for crizotinib-treated patients ranged from 53.13% to 85.32% and from 14.29% to 61.7%, respectively. Pooled analyses demonstrated significant improvements in both six-month and 12-month PFS relative to chemotherapy (OR = 2.84; 95% CI, 2.23-3.61; p < 0.00001 and OR = 4.33; 95% CI, 2.64-7.10; p < 0.00001, respectively). In contrast, although one- and two-year OS rates for crizotinib ranged from 27.5% to 97.1% and from 48.3% to 87.5%, no statistically significant differences in OS were observed when compared with chemotherapy at either time point (one-year OS: OR = 1.56; 95% CI, 0.81-2.99; p = 0.18; two-year OS: OR = 1.84; 95% CI, 0.95-3.58; p = 0.07). Overall, these findings indicate that while crizotinib confers substantial improvements in tumor response and disease control, its effect on long-term survival outcomes appears limited. Given the multifactorial determinants of OS, future investigations incorporating more granular patient stratification may be necessary to better delineate the role of crizotinib within the evolving treatment landscape for ALK-positive NSCLC.

## Introduction and background

Lung cancer remains the most common cause of cancer-related mortality globally, with non-small cell lung cancer (NSCLC) accounting for approximately 85% of cases. Mortality projections for 2025 indicate that lung cancer will remain disproportionately lethal compared to other malignancies, contributing to nearly 20% of all cancer deaths, compared with 8.56% for colorectal and 8.41% for pancreatic cancers [1]. Recent advances in molecular oncology have identified several oncogenic drivers and corresponding therapeutic targets that have transformed NSCLC management.

One such target is the anaplastic lymphoma kinase (ALK) gene, a receptor tyrosine kinase whose rearrangements occur in approximately 3-5% of NSCLC cases, most commonly as EML4-ALK fusions [2]. ALK rearrangements are commonly identified using immunohistochemistry (IHC), often with confirmatory fluorescence in situ hybridization (FISH), or increasingly via next-generation sequencing (NGS) panels [2]. Identification of ALK rearrangements led to the development of crizotinib, a small-molecule inhibitor of ALK, ROS1, and MET kinases [3,4]. The pivotal phase I trial, PROFILE 1001, established crizotinib as a promising targeted agent, reporting an objective response rate (ORR) of approximately 57% in ALK-positive NSCLC [5].

Despite significant early success, subsequent clinical experience revealed important limitations of crizotinib. Its poor central nervous system (CNS) penetration contributes to a high incidence of intracranial progression, as the CNS frequently serves as a sanctuary site for relapse [6,7]. Furthermore, secondary ALK mutations (e.g., L1196M, G1269A, G1202R) and activation of bypass signaling pathways such as EGFR and KIT have been implicated in acquired resistance [8-11]. While crizotinib consistently improves progression-free survival (PFS) and tumor response, its effect on overall survival (OS) remains variable across clinical trials and real-world studies [12-14].

Although second- and third-generation ALK inhibitors now dominate first-line management, crizotinib remains an important reference standard for benchmarking early ALK-targeted therapy and for interpreting the observed disconnect between disease control (ORR/PFS) and long-term survival outcomes (OS), which may be influenced by treatment crossover and subsequent therapies. Given its historical significance as the first approved ALK inhibitor, a comprehensive synthesis of its therapeutic efficacy remains clinically relevant. This meta-analysis aims to evaluate ORR, PFS, and OS outcomes associated with crizotinib therapy in ALK-positive NSCLC and to contextualize its clinical role relative to chemotherapy and next-generation ALK inhibitors.

## Review

Methods

Literature Search Strategy

The aim of this study is to achieve a better understanding of the clinical efficacy of crizotinib using peer-reviewed studies published in core clinical journals. A systematic search of the PubMed database was conducted using various combinations of keywords, including “crizotinib”, “lung cancer”, “response”, and “survival”. The literature search and study selection procedures were conducted in accordance with Preferred Reporting Items for Systematic Reviews and Meta-Analysis (PRISMA) guidance and aligned with Cochrane best-practice standards [15].

Population, Intervention, Comparison, and Outcomes (PICO) Framework

The inclusion criteria for this meta-analysis were defined using the PICO model (Table 1).

**Table 1:** Population, Intervention, Comparison, and Outcomes framework for study inclusion in the crizotinib meta-analysis Eligible studies included patients with confirmed ALK-positive NSCLC treated with first-line crizotinib monotherapy and compared against chemotherapy regimens. Outcomes extracted included ORR, PFS, and OS, with emphasis on time-specific endpoints (six- and 12-month PFS; one- and two-year OS). ALK: anaplastic lymphoma kinase; NSCLC: non-small cell lung cancer

PICO Element	Definition
Population	Patients with ALK-positive lung cancer treated with crizotinib. Studies exclusively evaluating brain metastases were excluded due to known limited CNS activity. No restrictions were placed on age, ethnicity, gender, or country of origin of studies.
Intervention	First-line therapy crizotinib monotherapy for ALK-positive NSCLC, irrespective of mutation subtype or dosing regimen.
Comparison	Chemotherapy regimens used as comparator arms.
Outcomes	Objective response rate (ORR), progression-free survival (PFS), overall survival (OS), including time-specific endpoints (six- and 12-month PFS; one- and two-year OS).

Study Selection

Inclusion criteria included, but were not limited to, intervention with crizotinib as monotherapy, chemotherapy as a comparison, report of at least one survival endpoint (PFS, ORR, or OS), and peer-reviewed. Pre-clinical studies, case reports, secondary analyses from previously published clinical trials, reviews, and meta-analyses were excluded. Studies without objective evidence of ALK alterations or crizotinib treatment were excluded as well. Additionally, only one set of findings from series studies based on the same patient populations was included, with preference given to the most recent and comprehensive dataset to avoid duplication of outcomes.

Risk of Bias Assessment

Risk of bias was evaluated only for studies that were included in the final meta-analysis. Randomized controlled trials (RCTs) were evaluated with the Cochrane Risk of Bias 2 (RoB 2) tool. Non-randomized studies were assessed using the Risk of Bias in Non-randomized Studies of Interventions (ROBINS-I) tool. Studies were categorized as low, moderate, or high risk of bias based on overall domain ratings, and these assessments were incorporated into the interpretation of pooled results.

Data Extraction, Statistical Analysis, and Sensitivity Analysis

Treatment regimens and clinical outcomes (ORR, PFS, OS) were gathered from studies that met the inclusion criteria and stored. PFS at six and 12 months, and OS at one and two years, were either directly extracted or estimated manually from published Kaplan-Meier curves when not explicitly stated. Pooled ranges of clinical outcomes from first-line crizotinib monotherapy treatment were reported. To compare the clinical efficacy of crizotinib and conventional chemotherapy, only results from first-line treatment were used. Meta-analysis was performed using Review Manager (RevMan) 5 (The Cochrane Collaboration, London, England, UK). Odds ratios (OR) were used to compare the outcomes of ORR, PFS, and OS rate at the time mentioned previously. Statistical significance was defined as p < 0.05. Sensitivity meta-analysis was performed by excluding studies marked as “high risk” or “serious risk” of bias according to RoB2 and ROBINS-I tool assessments.

Results

Studies Screening and Data Extraction

**Figure 1:**
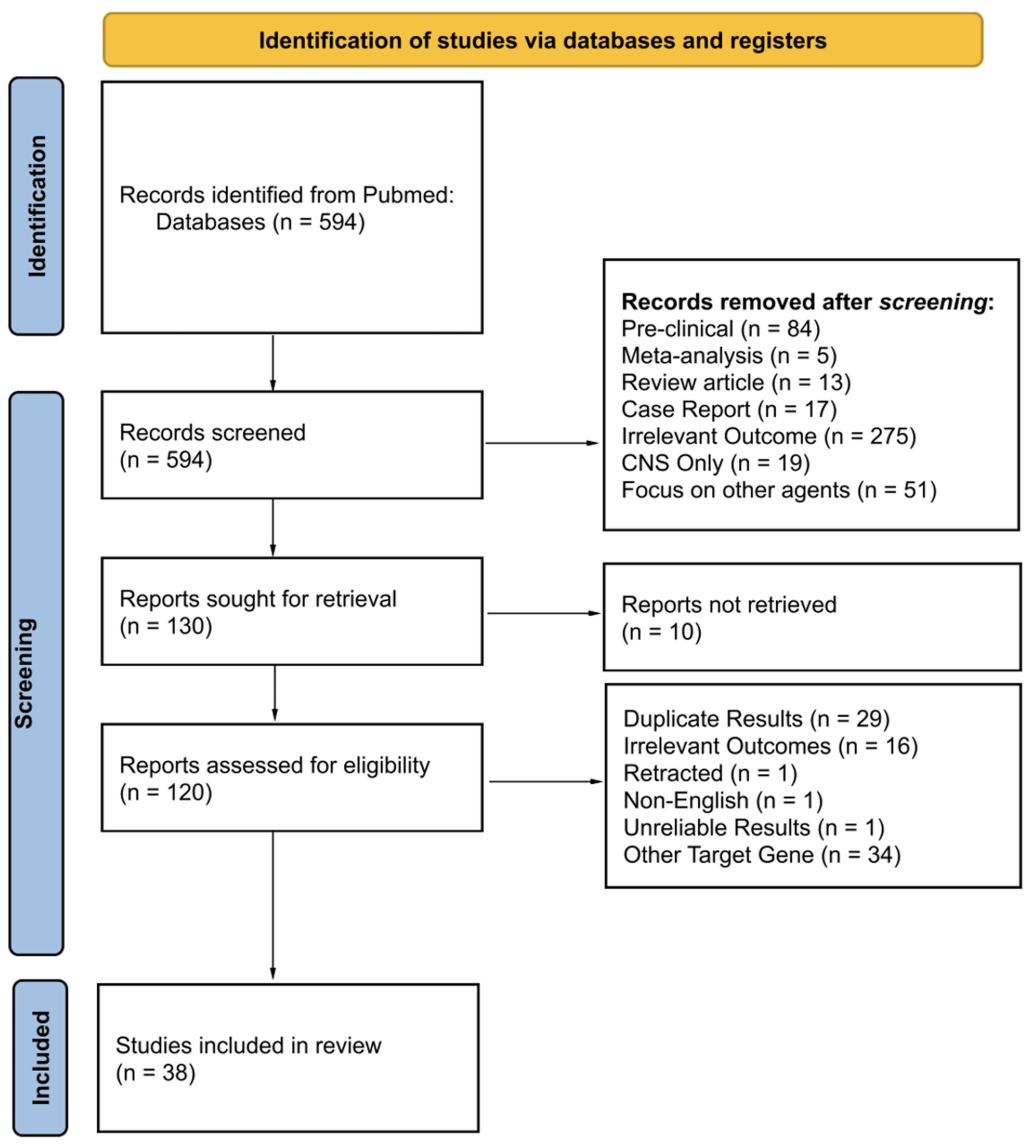
Preferred Reporting Items for Systematic Reviews and Meta-Analyses (PRISMA) flow diagram of study identification, screening, and inclusion

Initial search yielded 594 studies, which were sequentially narrowed to 38 studies that meet inclusion criteria. Figure 1 demonstrates the PRISMA flowchart of study selection [15].

Objective Response Rate (ORR)

**Figure 2:**
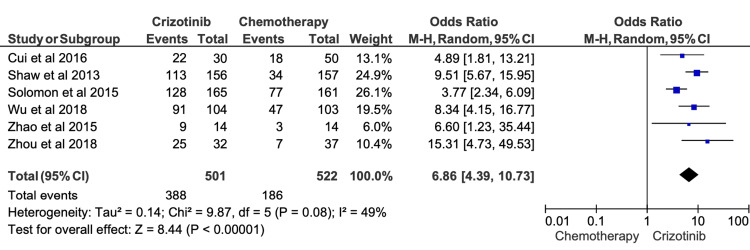
Comparative meta-analysis of objective response rate (ORR) for crizotinib versus chemotherapy Data extracted from: [26,12,19,16,32,18]

ORR was extracted from 26 studies, demonstrating that patients receiving crizotinib achieved an ORR ranging 50.2-87.0% (Table 2). Six studies were eligible for quantitative synthesis. Meta-analysis showed that crizotinib was associated with a significantly higher ORR compared with chemotherapy (OR = 6.86; 95% CI, 4.39-10.73; p < 0.00001; Figure 2). Moderate heterogeneity was observed among the included studies (I^2^ = 49%).

**Table 2:** Objective response rates (ORRs) for crizotinib in ALK-positive non-small cell lung cancer across included studies

Study (Author, Year)	Patients (n)	Responses (n)	ORR (%)
Wu 2018 [16]	104	91	87.5
Zhou 2019 [17]	60	48	80
Zhou 2018 [18]	32	25	78.1
Solomon 2015 [19]	165	128	77.6
Camidge 2019 [20]	148	114	77
Huang 2019 [21]	31	23	74.2
Yang 2023 [22]	129	94	72.9
Shaw 2013 [12]	156	113	72.4
Noronha 2016 [23]	52	37	71.2
Lei 2015 [24]	61	43	70.5
Hida 2017 [25]	104	73	70.2
Cui 2016 [26]	56	39	69.6
Asao 2017 [27]	13	9	69.2
Kilickap 2024 [28]	329	227	69
Solomon 2024 [29]	137	92	67.2
Wang 2022 [30]	109	73	67
Ito 2016 [31]	27	18	66.7
Zhao 2015 [32]	14	9	64.3
Li 2018 [33]	95	61	64.2
Tian 2020 [34]	41	26	63.4
Deng 2019 [35]	47	29	61.7
Wu 2015 [36]	21	13	61.9
Camidge 2012 [37]	143	87	60.8
Jeon 2023 [38]	258	155	60.1
de la Rosa 2022 [39]	91	48	52.7
Duruisseaux 2017 [14]	267	134	50.2

Six-Month PFS

**Figure 3:**
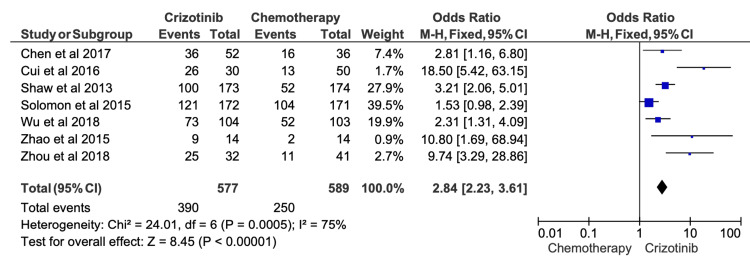
Comparative meta-analysis of six-month progression-free survival (PFS) for crizotinib versus chemotherapy Data extracted from: [42,26,12,19,16,32,18]

Across 28 included studies, six-month PFS rates among patients receiving first-line crizotinib ranged from 53.1% to 85.3% (Table 3). When compared with chemotherapy, meta-analysis demonstrated that crizotinib was associated with a significant improvement in six-month PFS (OR = 2.84; 95% CI, 2.23-3.61; p < 0.00001) (Figure 3). Substantial heterogeneity was observed among contributing studies (I^2^ = 75%), likely reflecting differences in patient populations, prior treatments, and study design.

**Table 3:** Six-month progression-free survival (PFS) outcomes following first-line crizotinib therapy in ALK-positive NSCLC ALK: anaplastic lymphoma kinase; NSCLC: non-small cell lung cancer

Study (Author, Year)	Patients (n)	Progression Free at Six Months (n)	Six-Month PFS (%)
Wang 2022 [30]	109	93	85.3
Wang 2023 [40]	47	39	82.9
Li 2018 [33]	96	79	82.3
Deng 2019 [35]	47	38	78.7
Zhou 2018 [18]	32	25	78.1
Kilickap 2024 [28]	329	257	78.1
Yang 2023 [22]	133	101	75.9
Su 2019 [41]	110	83	75.5
de la Rosa 2022 [39]	91	67	73.6
Tian 2020 [34]	41	30	73.2
Noronha 2016 [23]	69	50	72.5
Huang 2019 [21]	35	25	71.4
Solomon 2015 [19]	172	121	70.4
Wu 2018 [16]	104	73	70.2
Jeon 2023 [38]	290	203	70
Camidge 2012 [37]	143	100	69.9
Chen 2017 [42]	52	36	69.2
Mok 2020 [43]	151	104	68.9
Zhou 2019 [17]	62	42	67.7
Solomon 2024 [29]	147	99	67.4
Cai 2021 [44]	37	24	64.9
Zhao 2015 [32]	14	9	64.3
Hida 2017 [25]	104	65	62.5
Ito 2016 [31]	31	19	61.3
Chayab 2024 [45]	80	48	60
Camidge 2021 [46]	138	80	58
Shaw 2013 [12]	173	100	57.8
Cha 2016 [47]	32	17	53.1

Twelve-Month PFS

**Figure 4:**
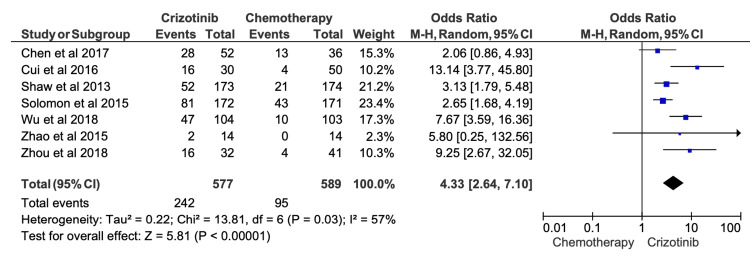
Comparative meta-analysis of one-year progression-free survival (PFS) for crizotinib versus chemotherapy Data extracted from: [42,26,12,19,16,32,18]

Twelve-month PFS outcomes showed greater variability, with reported rates ranging from 14.3% to 61.7% across included studies (Table 4). Despite this variability, pooled analysis demonstrated that crizotinib remained significantly superior to chemotherapy at the 12-month time point (OR = 4.33; 95% CI, 2.64-7.10; p < 0.00001) (Figure 4). Moderate heterogeneity was observed in the 12-month PFS meta-analysis (I^2^ = 57%), suggesting a more consistent relative benefit at later time points compared with six-month outcomes.

**Table 4:** Twelve-month progression-free survival outcomes following first-line crizotinib therapy in ALK-positive NSCLC ALK: anaplastic lymphoma kinase; NSCLC: non-small cell lung cancer

Study (Author, Year)	Patients (n)	Progression Free at One Year (n)	One-Year PFS (%)
Deng 2019 [35]	47	29	61.7
de la Rosa 2022 [39]	91	54	59.3
Kilickap 2024 [28]	329	193	58.7
Huang 2019 [21]	35	19	54.3
Jeon 2023 [38]	290	157	54.1
Chen 2017 [42]	52	28	53.9
Wang 2022 [30]	109	58	53.2
Zhou 2018 [18]	32	16	50
Solomon 2015 [19]	172	81	47.1
Wu 2018 [16]	104	47	45.2
Zhou 2019 [17]	62	28	45.2
Camidge 2012 [37]	143	64	44.8
Yang 2023 [22]	133	58	43.6
Mok 2020 [43]	151	65	43.1
Wang 2023 [40]	47	20	42.6
Cai 2021 [44]	37	15	40.5
Su 2019 [41]	110	40	36.4
Camidge 2021 [46]	138	46	33.3
Noronha 2016 [23]	69	23	33.3
Solomon 2024 [29]	147	48	32.7
Chayab 2024 [45]	80	26	32.5
Tian 2020 [34]	41	13	31.7
Shaw 2013 [12]	173	52	30.1
Cha 2016 [47]	32	9	28.1
Li 2018 [33]	96	27	28.1
Ito 2016 [31]	31	7	22.6
Hida 2017 [25]	104	21	20.2
Zhao 2015 [32]	14	2	14.3

One-Year OS

**Figure 5:**
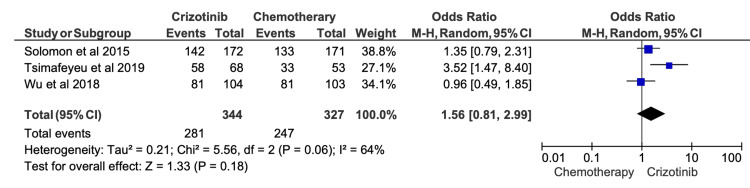
Comparative meta-analysis of one-year overall survival (OS) for crizotinib versus chemotherapy Data extracted from: [19,52,16]

One-year OS rates were extracted from 14 studies, with reported survival among patients receiving crizotinib ranging from 27.5% to 97.1% (Table 5). When compared with chemotherapy, pooled meta-analysis demonstrated a numerical survival advantage for crizotinib; however, this difference did not reach statistical significance (OR = 1.56; 95% CI, 0.81-2.99; p = 0.18) (Figure 5). Substantial heterogeneity was observed among included studies (I^2^ = 64%), likely reflecting variability in patient characteristics, follow-up duration, treatment crossover, and subsequent lines of therapy.

**Table 5:** Pooled one-year overall survival for patients receiving crizotinib

Study (Author, Year)	Patients (n)	Alive at One Year (n)	One-Year Overall Survival (%)
Hotta 2022 [48]	104	101	97.1
Noronha 2016 [23]	69	64	92.8
Wang 2023 [40]	47	43	91.5
Su 2019 [41]	110	99	90
Camidge 2021 [46]	138	116	84.1
Solomon 2018 [49]	172	142	82.6
Wu 2018 [16]	104	81	77.9
de la Rosa 2022 [39]	91	70	76.9
Camidge 2012 [37]	143	107	74.8
Mok 2020 [43]	151	104	68.9
Harada 2021 [50]	9	6	66.7
Chayab 2024 [45]	80	53	66.3
Duruisseaux 2017 [14]	318	179	56.3
Cao 2014 [51]	40	11	27.5

Two-Year OS

**Figure 6:**
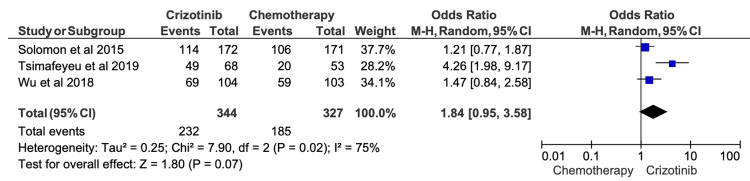
Comparative meta-analysis of two-year overall survival (OS) for crizotinib versus chemotherapy Data extracted from: [19,52,16]

Two-year OS outcomes were reported in 11 studies, with survival rates ranging from 48.3% to 87.5% among crizotinib-treated patients (Table 6). Meta-analysis similarly suggested a trend toward improved survival with crizotinib compared with chemotherapy; however, this finding did not achieve statistical significance (OR = 1.84; 95% CI, 0.95-3.58; p = 0.07) (Figure 6). Considerable heterogeneity persisted at the two-year time point (I^2^ = 75%).

**Table 6:** Pooled two-year survival for patients receiving crizotinib OS: overall survival

Study (Author, Year)	Patients (n)	Alive at Two Years (n)	Two-Year OS (%)
Hotta 2022 [48]	104	91	87.5
Noronha 2016 [23]	69	56	81.2
Wang 2023 [40]	47	37	78.7
Su 2019 [41]	110	75	68.2
Camidge 2021 [46]	138	94	68.1
de la Rosa 2022 [39]	91	61	67.0
Harada 2021 [50]	9	6	66.7
Wu 2018 [16]	104	69	66.3
Solomon 2018 [49]	172	114	66.3
Chayab 2024 [45]	80	44	55
Mok 2020 [43]	151	73	48.3

Risk of Bias Analysis

**Figure 7:**
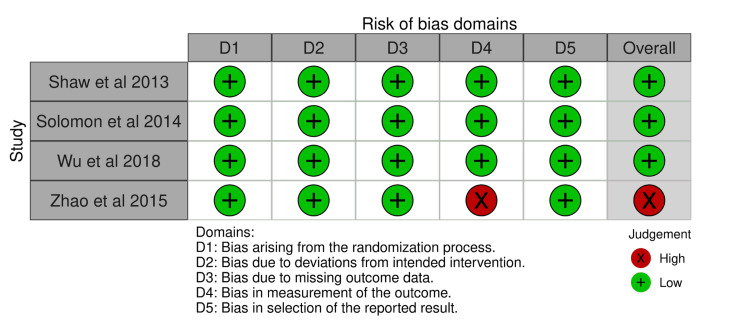
Risk of bias evaluation for included randomized controlled trials using the Cochrane Risk of Bias 2 (ROB2) tool Included studies: [12,29,16,32] The traffic-light visualization plot was generated using the Robvis tool for risk-of-bias visualization [53].

The ROB2 assessments demonstrated that the included RCTs generally had low risk of bias across all domains, with the exception of one study (Zhao et al. 2015), which showed high risk in the domain related to outcome measurement (Figure 7). Overall, the RCTs were considered methodologically robust and unlikely to substantially distort effect estimates.

**Figure 8:**
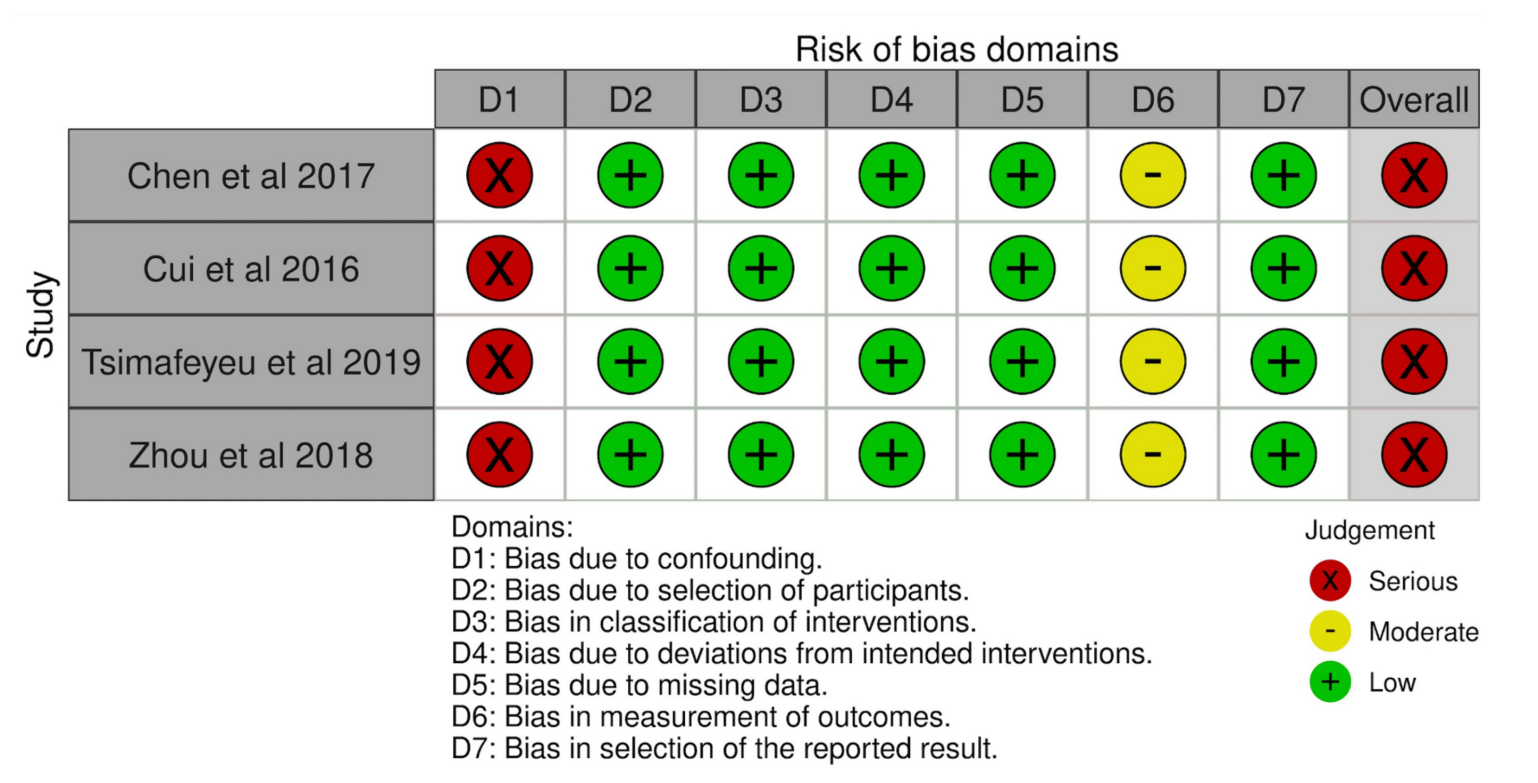
Risk of bias evaluation for included non-randomized observational studies using the Risk of Bias in Non-randomized Studies of Interventions (ROBINS-I) tool Included studies: [42,26,51,18] The traffic-light visualization plot was generated using the Robvis tool for risk-of-bias visualization [53].

In contrast, the ROBINS-I evaluations revealed that all retrospective studies carried a serious risk of bias in Domain 1 (bias due to confounding) (Figure 8). As expected, retrospective observational designs lack randomized assignment and therefore cannot reliably account for all confounding variables. For example, patients receiving crizotinib versus chemotherapy may differ systematically in baseline characteristics, disease severity, comorbidities, prior treatments, or molecular status. As a result, retrospective studies were categorized as having a serious overall risk of bias, despite low or moderate concerns in subsequent domains such as participant selection, classification of interventions, and missing data.

Sensitivity Analysis

**Figure 9:**
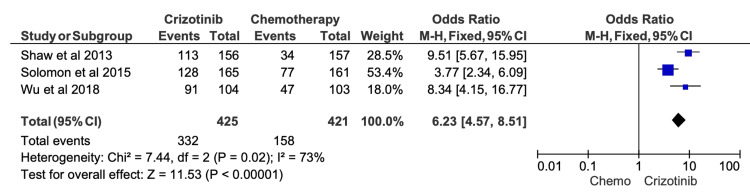
Comparative meta-analysis of objective response rate results after excluding studies rated as high or serious risk of bias After restricting the analysis to studies with acceptable risk-of-bias ratings, crizotinib remained associated with a significantly higher objective response rate compared with chemotherapy (OR = 6.23; 95% CI, 4.57-8.51; p < 0.00001). Included studies: [12,19,16]

**Figure 10:**
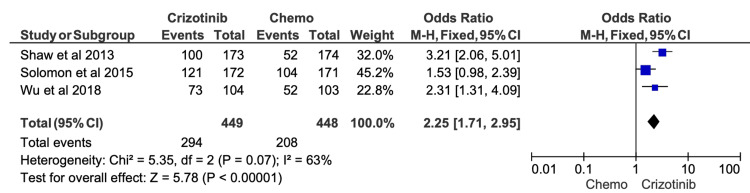
Comparative meta-analysis of six-month progression-free survival (PFS) for crizotinib versus chemotherapy after excluding studies rated as high or serious risk of bias After restricting the analysis to studies with acceptable risk-of-bias ratings, crizotinib remained associated with a significantly higher six-month PFS compared with chemotherapy (OR = 2.25; 95% CI, 1.71-2.95; p < 0.00001). Included studies: [12,19,16]

**Figure 11:**
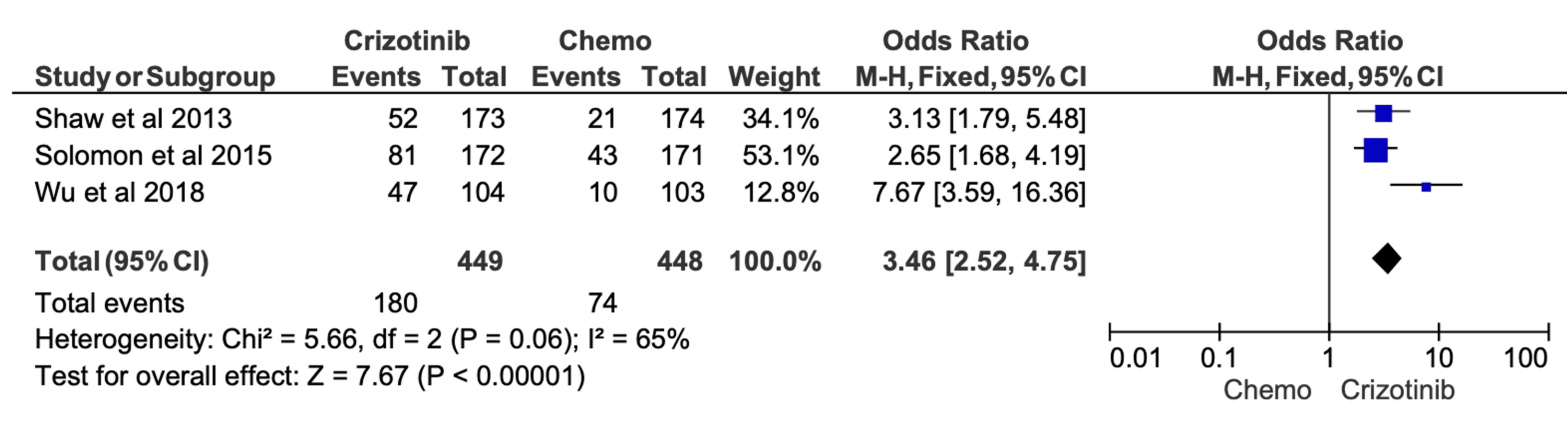
Comparative meta-analysis of 12-month progression-free survival (PFS) for crizotinib versus chemotherapy after excluding studies rated as high or serious risk of bias After restricting the analysis to studies with acceptable risk-of-bias ratings, crizotinib remained associated with a significantly higher 12-month PFS compared with chemotherapy (OR = 3.46; 95% CI, 2.52-4.75; p < 0.00001). Included studies: [12,19,16]

**Figure 12:**
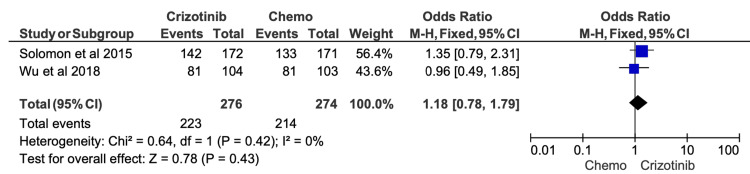
Comparative meta-analysis of one-year overall survival (OS) for crizotinib versus chemotherapy after excluding studies rated as high or serious risk of bias After restricting the analysis to studies with acceptable risk-of-bias ratings, crizotinib demonstrated no significant difference in one-year OS compared with chemotherapy (OR = 1.18; 95% CI, 0.78-1.79; p = 0.43). Included studies: [19,16]

**Figure 13:**
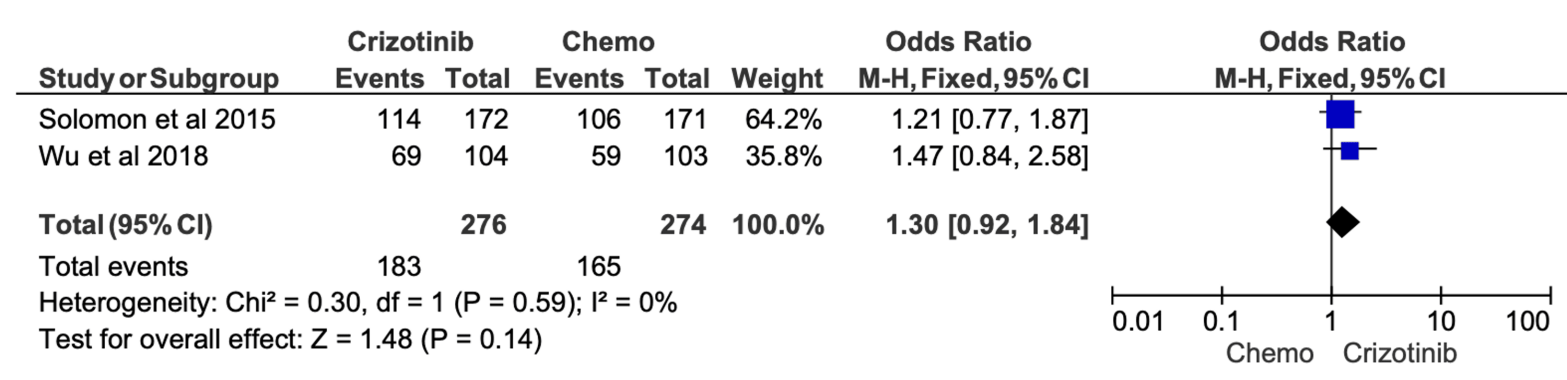
Comparative meta-analysis of two-year overall survival (OS) for crizotinib versus chemotherapy after excluding studies rated as high or serious risk of bias After restricting the analysis to studies with acceptable risk-of-bias ratings, crizotinib demonstrated no significant difference in two-year OS compared with chemotherapy (OR = 1.30; 95% CI, 0.92-1.84; p = 0.14). Included studies: [19,16]

Given the high risk of confounding variables in retrospective studies, we conducted sensitivity analyses excluding all studies flagged as “serious risk” in their overall ROB assessments. After removal of these studies, the effect sizes for ORR, six-month PFS, 12-month PFS, and OS were recalculated (Figures 9-13). The findings remained directionally consistent with the primary analyses.

Discussion

This systematic review and meta-analysis of 38 included studies demonstrated that crizotinib significantly improves ORR and PFS with no significant improvement in OS compared to chemotherapy in patients with ALK-NSCLC. Analysis revealed that patients receiving crizotinib had over six times higher response to crizotinib than chemotherapy. Patients treated with crizotinib had approximately three-fold increased odds at six-months and over three and a half times higher odds at 12 months of remaining progression-free. However, OS outcomes were inconsistent, with no statistically significant difference at one or two years between crizotinib and chemotherapy, even though a slight improvement was seen in crizotinib-treated patients. High study heterogeneity was observed in the pooled analysis for six- and 12-month PFS as well as one- and two-year OS, suggesting that the magnitude of benefit varied across studies. This variability may reflect the differences in patient characteristics and comparison regimens. These results demonstrate a clinical paradox of substantial short-term disease control without long-term survival benefit.

The biological activity of crizotinib can be explained by its targeted inhibition of tyrosine kinases ALK, ROS1, and MET. In ALK-positive NSCLC, inhibition of the EML4-ALK fusion protein suppresses downstream proliferative pathways, including PI3K/AKT, MAPK/ERK, and JAK/STAT [15]. The molecular blockade accounts for the robust improvements in ORR and PFS across clinical trials [53]. Despite these advantages in ORR and PFS, several intrinsic limitations restrict crizotinib’s long-term survival benefit. Most notably, crizotinib’s poor penetration of the CNS allows for CNS metastasis, a common pattern of progression [6]. Other mechanisms, including secondary ALK mutations such as L1196M, G1269A, and G1202R, allow for ALK amplification and bypass signaling pathways, diminishing the durability of crizotinib therapy [8].

The findings presented in this study mirror results from other RCTs, such as the PROFILE 1007 and PROFILE 1014 studies. These studies demonstrated improved ORR and PFS compared to chemotherapy but little benefit on OS [12,19]. Other real-world cohort and retrospective studies have also demonstrated that crizotinib may provide benefits in ORR and PFS with little benefit on OS, suggesting the need for a wider acceptance of next-generation small molecule inhibitors [13,14].

The clinical implication of this meta-analysis evaluating crizotinib versus chemotherapy is twofold. First, crizotinib, as the first approved targeted therapy, remains an important historical milestone in treating ALK-positive NSCLC and paved the way for targeted treatment for lung cancer. Second, the observed limitations in OS explain why next-generation ALK inhibitors such as alectinib, brigatinib, and lorlatinib have supplementary value compared to crizotinib, given their superior efficacy and greater penetrance of the CNS [29,45,54].

This study is not without limitations. High rates of treatment crossover confound OS analyses, and variability in follow-up duration may influence reported OS rates. Additionally, subgroup analyses by age, sex, and comorbidities were not consistently reported, limiting the ability to assess similarities in studies. Despite these limitations, the consistency of findings across a large number of studies strengthens the reliability of our findings.

Due to high risk of bias as assessed by the ROBINS-I tool, sensitivity analyses were performed, which indicate that while retrospective studies introduced substantial confounding risk, the overall conclusions regarding short-term treatment efficacy (ORR and PFS) remained robust. While the OS analyses remained nonsignificant after removal of high-risk studies, this underscores the limitations of the available evidence regarding long-term survival outcomes.

## Conclusions

In summary, this meta-analysis demonstrates that while crizotinib markedly improves tumor response and delays progression in ALK-positive NSCLC, its effect on OS is limited. These results highlight both the strengths and limitations of first-generation ALK inhibitors and support the clinical consideration of next-generation agents when long-term outcomes are a priority.
